# SRS/SBRT commissioning process with a hybrid beam data model in new preconfigured CyberKnife system

**DOI:** 10.1002/acm2.70822

**Published:** 2026-09-25

**Authors:** Mikoto Tamura, Yasumasa Nishimura, Tomohiro Matsuura, Yuta Inosaka, Yoshiyuki Sadahira, Ayano Akamine, Kyomi Inoue, Seiki Nishino, Hirokazu Mizuno, Iori Sumida, John M. Noll

**Affiliations:** ^1^ Department of Radiation Therapy and Medical Physics Izumiotsu Medical Center Izumiotsu Osaka Japan; ^2^ Radiation Therapy Center Izumiotsu Medical Center Izumiotsu Osaka Japan; ^3^ Accuray Japan K.K., Otemachi Chiyoda‐ku Tokyo Japan; ^4^ Accuray Inc. Sunnyvale California USA

**Keywords:** CyberComm, CyberKnife, Hybrid beam data model, SRS/SBRT commissioning

## Abstract

**Background:**

A new preconfigured CyberKnife system was introduced to improve the SRS/SBRT commissioning efficiency. The vendor provides a reference beam data set, termed “beam data model (BDM)”, and recommends its validation and combination with measurement data to develop a hybrid beam data model (h‐BDM). However, the validation method by the user and the clinical acceptability with the h‐BDM has not been established.

**Purpose:**

This study reports the first clinical validation of the BDM and aims to demonstrate the SRS/SBRT commissioning process with the h‐BDM is clinically acceptable.

**Methods:**

The BDM was validated by comparing with the measurements of tissue phantom ratios (TPRs), off‐center ratios (OCRs), and output factors (OFs) for fixed and Iris collimators and MLC in CyberKnife S7 system. Dose difference (DD) and distance‐to‐agreement (DTA) between BDM and measurement data were assessed against predefined thresholds: DDs of 1.0% for TPRs; DDs of 1.0% at d_15mm_ and d_100mm_ and 1.5% at d_300mm_ for central regions in OCRs; and DTAs of 0.3–1.0 mm for penumbra regions. The h‐BDM was constructed by supplementing the BDM with the measurement beam data for all OFs, and for the TPRs and OCRs of the three smallest field sizes with each collimator. Additional replacements were also made when discrepancies exceeded the proposed thresholds based on clinical usability considerations. The conventional measurement beam data model (m‐BDM) was also developed with only measurement data. Single‐beam validation was performed in homogeneous water‐equivalent phantom and heterogeneous phantoms, including lung‐equivalent slabs, by comparing between the measured and calculated point doses with h‐BDM and m‐BDM at multiple depths. Finally, patient‐specific quality assurance (PSQA) was performed for SRS/SBRT plans. In phantom case, SRS/SBRT plans were created for a single spherical target on the CT images of the StereoPHAN phantom and anthropomorphic lung phantom for brain and lung sites, respectively. For the SBRT plans of spine and prostate sites, the C‐shape and test‐prostate structure sets, provided by the AAPM TG 119, were employed. In clinical case, six SRS/SBRT plans for each patients’ CT images in brain, lung, spine, and prostate sites, were employed and the PSQA plans were generated using the CT images of the StereoPHAN phantom, anthropomorphic lung phantom, and the I'mRT phantom. The point dose and γ pass rate were evaluated using micro ionization chamber and radiochromic film, respectively.

**Results:**

Differences between the BDM and measurement exceeded the specific thresholds for the OCRs with three largest MLC field sizes. In the single‐beam validation, dose differences between measurements and calculations with h‐BDM were within 2.0% for field sizes of > 10.0 mm, except up to 3.0% at 140 mm depth with MLC, and within 5.0% for field sizes of ≤10.0 mm. In PSQA with the h‐BDMs, the point dose differences were within 3.0% and the γ pass rates (2%/2 mm and 3%/1 mm) were > 95.0% for all SRS/SBRT plans. The single‐beam validation and PSQA results of the h‐BDM were comparable to those of the conventional m‐BDM.

**Conclusions:**

The SRS/SBRT commissioning process with the proposed h‐BDM provides a new clinically acceptable framework.

## 1 INTRODUCTION

The CyberKnife System (Accuray Inc., Madison, WI) can deliver the stereotactic radiosurgery (SRS) and stereotactic body radiotherapy (SBRT) with submillimeter accuracy.^1^, ^2^ To achieve the accurate and efficient SRS/SBRT, the treatment planning system (TPS) must be adequately commissioned. The commissioning process is inherently complex and time‐consuming, demanding meticulous beam data collection, robust beam modelling and rigorous model validation.^3^ Errors introduced during commissioning can compromise dosimetric accuracy, consequently patient outcome, and carry the risk of a negative incident potentially.^4^ Several studies have also reported large inter‐unit variations in the beam data of the CyberKnife System, especially for extremely small irradiation fields. This is due to measurement difficulties by the lack of lateral electron equilibrium and high dose gradients and considerable inter‐detector variability such as the ion‐chamber, diode, and synthetic diamond.^5^, ^6^, ^7^, ^8^, ^9^

To reduce the data acquisition burden, the CyberComm method of twinning CyberKnife System linear accelerators (linacs) to a standardized beam model was introduced (Accuray Inc.). First, beam twinning was performed by the vendor for more than ten CyberKnife linacs referenced to a single original unit by tuning each system with a Semiflex3D chamber (PTW, Freiburg, Germany) in a three‐dimensional water tank to match the reference beam characteristics with and without the secondary collimator systems, including the fixed collimator, Iris variable aperture collimator (Iris collimator), and InCise2 multileaf collimator (MLC). Each twinned linac then underwent full commissioning using a PTW 60019 microDiamond detector (PTW). The tissue phantom ratios (TPRs), off‐center ratios (OCRs) at depths of 15, 100, and 300 mm (d_15mm_, d_100mm_, d_300mm_), and output factors (OFs) in water were measured. The vendor constructed a Beam Data Model (BDM) derived from more than ten linacs as a reference beam data. Users are advised to perform the validation of the BDM and supplement the excluded components—the three smallest field sizes for TPR and OCR datasets and all OFs across field sizes—through the direct measurements at their site following the vendor's beam twinning test with the γ pass rates. By integrating the validated BDM with these site‐specific measurements, hybrid beam data model (h‐BDM) is constructed for use in SRS/SBRT treatment planning and verification.^10^, ^11^

This preconfigured system is expected to mitigate the negative incident risk and workload in the SRS/SBRT commissioning process. However, the BDM validation method by the user and the clinical acceptability with the h‐BDM has not been established. The discrepancies between the reference and user‐measured beam data can also compromise the delivery accuracy of SRS/SBRT when the reference BDM is used. In particular, the reproducibility of manufacturing procedures, such as beam twinning process in the case of CyberComm, can affect the performance of the preconfigured system.^3^, ^12^ Furthermore, the calculated dose distribution in a clinical plan can be substantially influenced by the beam model even when the reference beam data is good agreement with the user‐measured beam data.^13^ The purpose of this study was to validate the BDM and to demonstrate the consequent SRS/SBRT commissioning with the h‐BDM was acceptable for clinical use. This is the first published account of the clinical implementation.

## 2 MATERIALS AND METHODS

This study comprised four steps: 1. Validation of the BDM through the comparison with the user‐measured beam data; 2. Development of the h‐BDM; 3. Single‐beam validation in homogeneous and heterogeneous phantom geometries; and 4. Patient‐specific quality assurance (PSQA) of SRS/SBRT plans in phantom and clinical cases.

### 2.1 Step 1: Validation of the BDM through the comparison with the measurement beam data

The beam data required for commissioning includes TPRs, OCRs, and OFs for 35 field sizes of 5.0, 7.5, 10.0, 12.5, 15.0, 20.0, 25.0, 30.0, 35.0, 40.0, 50.0, and 60.0 mmφ for fixed and Iris collimators and 7.6 × 7.7, 15.4 × 15.4, 23.0 × 23.1, 30.8 × 30.8, 38.4 × 38.5, 46.2 × 46.2, 53.8 × 53.9, 69.2 × 69.3, 84.6 × 84.7, 100.0 × 100.1, and 115.0 × 100.1 mm^2^ for MLC (X × Y). The PTW 60019 microDiamond detector and SunSCAN 3D water tank (Sun Nuclear Corp., Melbourne, FL) were employed in all beam data measurements. The TPR was normalized using the d_15mm_ value. The OCRs at d_15mm_, d_100mm_, and d_300mm_ were obtained at a source‐to‐surface distance (SSD) of 800 mm. The OFs at d_15mm_ for each collimator were obtained with an SSD of 785 mm. This was defined as the ratio to the dose at d_15mm_ with a fixed 60.0 mmφ collimator. The correction factors for the OFs were not applied, consistent with the BDM. Then, the measured TPRs and OCRs for all but the three smallest fields for each collimator were compared with the BDM. The global dose difference (DD) was evaluated beyond the d_15mm_ for the TPR. The OCR was normalized at central axis (CAX) value and divided into three regions of central, shoulder, and penumbra at each depth. Two‐minima (M1 and M2, where M1 was closer to the center) of the third‐derivative profiles of the BDM by fitting with sigmoid function in Equation (1) were used to determine the divided regions.^14^

(1) $$S\left(r\right)=\beta_{1}-\frac{\beta_{2}}{1+\exp\left(\frac{r-\alpha_{1}}{\alpha_{2}}\right)}$$

where *r* is the radial distance from CAX and α_1_, α_2_, β_1_, and β_2_ are field size and depth dependent parameters. The central region was defined as the area extending from the central axis to 6.0 mm upstream of M1 and the shoulder region encompassed the area beyond this 6.0 mm boundary.^14^ In the central and shoulder regions, the global DDs were evaluated, while the distance‐to‐agreement (DTA) was evaluated in the penumbra region, defined as the area between the points of M1 and M2. The OF differences for each field size were also evaluated. The vendor verified the measured beam data using a twinning test with γ pass rates; however, the γ pass rate lacks sensitivity to the magnitude of discrepancies between the BDM and measurement data for user validation. Therefore, we proposed the thresholds of DDs of TPRs beyond the d_15mm_ and OCRs in the central region at d_15mm_, d_100mm_, and d_300mm_, and DTAs^11^ of OCRs in the penumbra region as a priori rationale, shown in Table 1.

**TABLE 1:** Proposed thresholds between vendor supplied beam data model (BDM) and measurement beam data for the dose differences (DDs) of TPR beyond the d_15mm_ and OCRs in the central region at d_15mm_, d_100mm_, and d_300mm_, and distance‐to‐agreement (DTA) of off‐center ratio (OCR) in the penumbra region.

			OCR		
		TPR	DD (%)		
Collimator	Field size	DD (%)	d_15mm_ & d_100mm_	d_300mm_	DTA (mm)
Fixed / Iris	12.5–20.0 mmφ	1.0	1.0	1.5	0.3
	25.0–60.0 mmφ				0.6
MLC	30.8 × 30.8 & 38.4 × 38.5 mm^2^	1.0	1.0	1.5	0.5
	46.2 × 46.2 & 53.8 × 53.9 mm^2^				0.6
	69.2 × 69.3 mm^2^				0.7
	84.6 × 84.7 mm^2^				0.8
	100.0 × 100.1 mm^2^				0.9
	115.0 × 100.1 mm^2^				1.0

*Note*: These thresholds differ from the vendor‐recommended values.

### 2.2 Step 2: Development of an h‐BDM

The h‐BDM was developed using Precision TPS (ver. 3.5.0, Accuray Inc., Madison, WI, USA). The beam data of the TPRs and OCRs for the three smallest field sizes (5.0, 7.5, and 10.0 mmφ for fixed and Iris collimators and 7.6 × 7.7, 15.4 × 15.4, and 23.0 × 23.1 mm^2^ for MLC) and all OFs were supplemented with the measurement data to the BDM according to the vendor's recommendation. When the differences between the BDM and measurement exceeded the thresholds given in Table 1, the beam data in the BDM were also replaced with the measurement beam data. The conventional measurement beam data model (m‐BDM) was also generated with only measurement data for comparison.

### 2.3 Step 3: Single‐beam validation in homogeneous and heterogeneous phantom geometries

**FIGURE 1:**
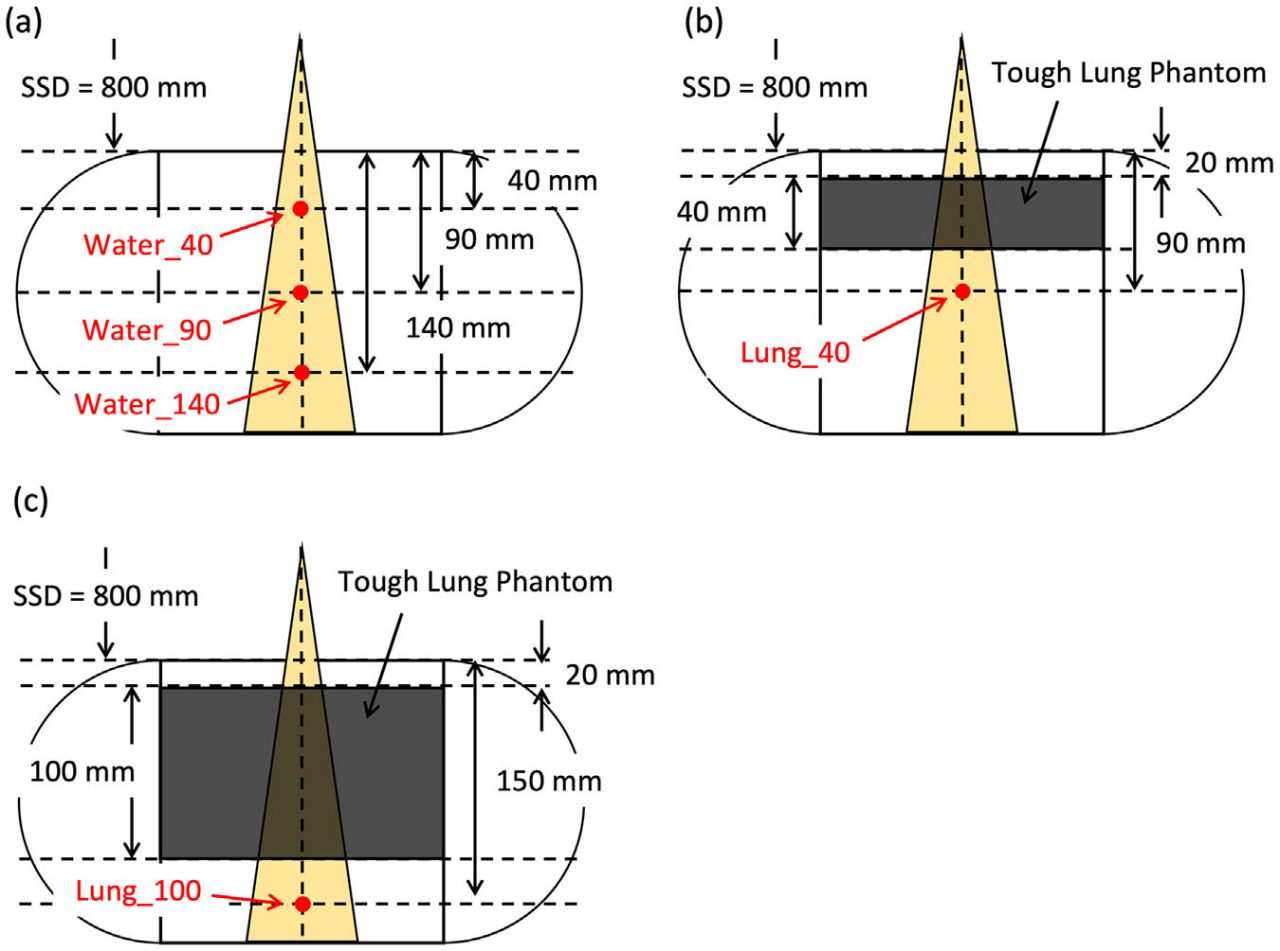
Homogeneous and heterogeneous phantom geometries for single‐beam validation. Measurement points were denoted as Water_40, Water_90, and Water_140 in homogeneous phantom and Lung_40 and Lung_100 in heterogeneous phantoms.

The calculated doses with the h‐BDM and m‐BDM were compared to the measured doses for the single‐beam using simple geometries in homogeneous and heterogeneous phantoms. These geometries were created using the I'mRT phantom (IBA Dosimetry, Schwarzenbruck, Germany) and tough lung slab phantom (Kyoto Kagaku Co., Ltd, Kyoto, Japan), as shown in Figure 1. The absorbed doses at each point were measured using the RAZOR ion‐chamber (IBA Dosimetry, Schwarzenbruck, Germany) that was cross‐calibrated using the Exradin A12S Farmer Type reference ion‐chamber (Standard Imaging, Madison, WI, USA) with the absorbed dose for the 60.0 mmφ beam of the fixed collimator at d_15mm_ in the three‐dimensional water tank. Each geometric phantom, containing the RAZOR ion‐chamber, was scanned using the SOMATOM go.Sim computed tomography (CT) system (Siemens Healthineers, Forchheim, Germany) with the scanning parameters set at 120 kV tube voltage, 500 mm field of view (FOV), and 1.0 mm slice thickness. The volume of the RAZOR ion‐chamber was delineated and assigned with the electron and mass densities of water in the TPS. The calculated dose was evaluated as the mean dose of the delineated volume of the RAZOR ion‐chamber. The ray tracing (RT) algorithm and the finite‐size pencil beam (FSPB) with lateral scaling correction calculation algorithms were employed with the homogeneous phantom geometry, while the Monte Carlo (MC) algorithm was employed with the heterogeneous phantom geometry. The size of the calculation grid was 0.98 × 0.98 × 1.00 mm^3^ and the desired relative statistical uncertainty for the MC algorithm was 0.5%. The field sizes studied for the fixed and Iris collimators were 5.0, 10.0, 20.0, 30.0, 40.0, and 60.0 mmφ (fixed) and 7.5, 10.0, 20.0, 30.0, 40.0, and 60.0 mmφ (Iris), while those for the MLC were 7.6 × 7.7, 23.0 × 23.1, 30.8 × 30.8, 46.2 × 46.2, 53.8 × 53.9, and 115.0 × 100.1 mm^2^. The irradiated monitor unit (MU) value was set as 100 MU. The calculated doses with each model were compared to the measured doses. Between‐model comparison of the calculated doses was also performed.

### 2.4 Step 4: PSQA of SRS/SBRT plans using the h‐BDM in phantom and clinical cases

To validate the deliverability of SRS/SBRT plans based on the h‐BDM, PSQA was performed with CT images from simulated tumors as phantom case and the patients as clinical case. To create the PSQA plans, the CT images of three phantom types of the StereoPHAN phantom (Sun Nuclear Corp., Melbourne, FL, USA), the anthropomorphic lung phantom (Dynamic thorax phantom, CIRS Inc., Norfolk, VA, USA), and the I'mRT phantom were employed. The phantoms were scanned using the SOMATOM go.Sim CT system, with scanning parameters of 120 kV tube voltage, 500 mm FOV, and 1.0 mm slice thickness.

In phantom case, the SRS/SBRT plans of four simulated treatment sites in the brain, lung, spine, and prostate were created using the h‐BDM for each CT image of each phantom. Single spherical target in the brain treatment site was delineated as gross tumor volume (GTV) at the center of the StereoPHAN phantom. The sizes of the simulated GTVs were 10.0, 20.0, and 30.0 mm diameters. The clinical target volume (CTV) was the same as the GTV and the planning target volume (PTV) margin of 1.0 mm was added to each CTV. The fractionated SRS plans with prescribed dose of 24 Gy/3 fr to D_99.5_ of the PTV were then created for the fixed and Iris collimators using the RT algorithm. For the 10.0 mmφ simulated GTV using the Iris collimator, the collimator size of 5.0 mmφ was employed as much as possible because the MU contribution of the 5.0 mmφ beam is limited to allow for a maximum expected error of 2.0% of the prescribed dose.^15^ For the lung site, each single spherical GTV target of 10.0, 20.0, and 30.0 mm diameters was delineated in the dynamic thorax phantom. The CTV was same volume to the GTV and the PTV was set by adding the margin of 5.0 mm to each CTV. SBRT plans were created for the MLC using the MC algorithm (the desired relative statistical uncertainty of 1.0%) with prescribed dose of 48 Gy/4 fr to D_95_ of the PTV. For the spine and prostate sites, the C‐shape and test‐prostate structure sets created by the AAPM TG 119^16^ were employed as the simulated target as PTV and organs at risk (OARs). The prescribed doses were 35 Gy/5 fr and 36.25 Gy/5 fr to D_95_ of each PTV for the spine and prostate sites, respectively. The plans were created with the MLC using the FSPB algorithm. All plans were created using the VOLO optimization to meet the goal of Paddick conformity index (CI) of > 0.80 for the PTV. The CI was defined as

(2) $$CI_{\mathrm{Paddick}}=\frac{{\mathrm{TV}}_{\mathrm{PIV}}^{2}}{\mathrm{TV}\times V_{\mathrm{RI}}},$$

where *TV* is the target volume, *TV_PIV_* is the target volume covered by the prescribed dose, and *V_RI_* is the total volume covered by the prescribed dose.^17^ The size of calculation grid was 0.98 × 0.98 × 1.00 mm^3^.

For the PSQA of the SRS/SBRT plans, experimental setup was summarized in Table 2. The point doses at the center of each GTV in the brain and lung sites and the arbitrary points in each PTV for the spine and prostate sites were measured using the RAZOR ion‐chamber, and these measurements were compared with each calculated dose. The differences of dose distributions were also evaluated by the global γ pass rate with three criteria in terms of DD and DTA of 2%/2 mm, 3%/1 mm, and 2%/1 mm with a threshold of 10.0% using the EBT4 Gafchromic film (Ashland ISP Advanced Materials, Bridgewater, NJ, USA). The film was inserted on the coronal, sagittal, and axial planes of each phantom at the relevant brain, lung, and spine and prostate sites. At least 24 h after exposure, the irradiated films were scanned using the Epson DS‐G30000 scanner (Epson Corp., Nagano, Japan) with a resolution of 75 dpi on the red channel. Optical density and dose calibration curve was created with a range from 0.0 to 10.0 Gy and the DoseLab ver. 7.0MR1 (Varian Medical Systems, Palo Alto, CA, USA) was employed for the analysis. The prescribed dose for the film dosimetry was scaled to a maximum of 6.0 Gy to meet the calibration curve range.^18^ The original SRS/SBRT plans created with the h‐BDM were copied and recalculated using the m‐BDM with the same calculation algorithms and delivery parameters, including nodes, beam numbers, beam directions, collimator sizes (fixed and Iris) or segment irradiation shapes (MLC), and MU. This ensured that any differences in the PSQA results were solely attributable to the used model. The point dose differences and γ pass rates were also compared between the models.

**TABLE 2:** Experimental setup of the patient‐specific quality assurance (PSQA) for the SRS/SBRT plans of four treatment sites in phantom case.

Treatment site	Target / Structure	Phantom	Collimator	Calculation algorithm
Brain	Spherical target (10.0, 20.0, and 30.0 mmφ)	StereoPHAN phantom	Fixed & Iris	RT
Lung	Spherical target (10.0, 20.0, and 30.0 mmφ)	Dynamic thorax phantom	MLC	MC
Spine	C‐Shape (AAPM TG119)	I'mRT phantom	MLC	FSPB
Prostate	Test prostate (AAPM TG119)	I'mRT phantom	MLC	FSPB

In clinical case, the CT images of the patients with treatment sites of brain, lung, spine, and prostate, who underwent the radiotherapy previously at our facility, were utilized. The Institutional Ethics Committee approved this study (institutional review board number: 2024018). The cases of pituitary neuroendocrine tumor (PitNET) and brain metastasis with a size of 14.0 mm were selected for the brain site and the fractionated SRS plans were created with the h‐BDM to meet the dosimetric goals as shown in Table S1, ^19^, ^20^, ^21^, ^22^, ^23^ with fixed and Iris collimators, respectively. For the lung site, the cases with tumor sizes of 19.0 and 38.0 mm were selected and for the spine site, thoracic spine metastasis case was selected. The SBRT plans for the lung, spine, and prostate cases were created with MLC and the dose distributions were calculated with h‐BDM to meet the dosimetric goals in Table S1.^19^, ^20^, ^21^, ^24^, ^25^, ^26^ Either RT or FSPB algorithm was employed for brain, spine, and prostate cases, while MC algorithm was employed for lung case.^27^ The PSQA plans were created by recalculation to each CT image of phantom with the same set of equipment to the phantom case for each treatment site, as shown in Table 2. As with the phantom cases, the h‐BDM based PSQA plans were copied and recalculated using the m‐BDM. PSQA of SRS/SBRT plans were implemented and the results of point dose difference and γ pass rate were also compared between the models. In the case where the beam data in the BDM were replaced with the measurement beam data because the differences between the BDM and measurement exceeded the thresholds given in Table 1, the additional pure h‐BDM was developed by supplementing the BDM with measurement beam data for all OFs and for TPRs and OCRs at only the three smallest field sizes, according to the vendor's recommendation. PSQA for the clinical case was also performed using the pure h‐BDM, and the results were compared with those obtained using the conventional m‐BDM.

## 3 RESULTS

### 3.1 Validation of the BDM through comparison with the measurement beam data

**FIGURE 2:**
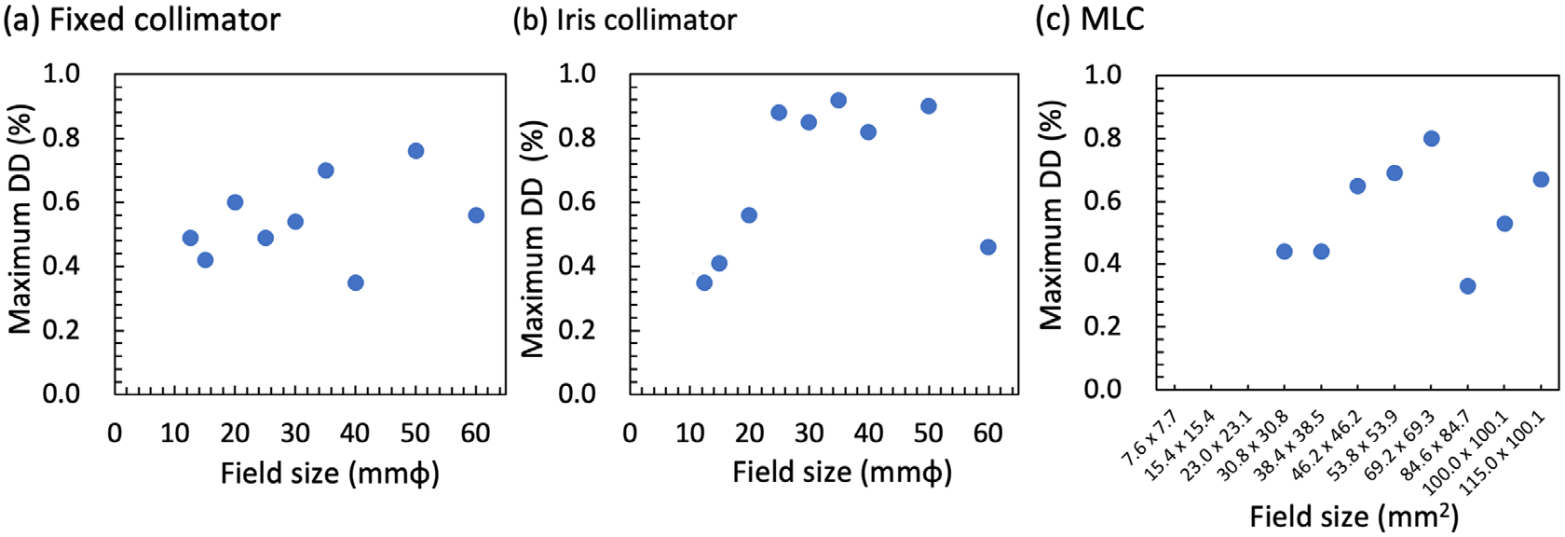
Maximum dose difference (DD) between the vendor supplied beam data (BDM) and measurement beam data for each tissue phantom ratio (TPR). The threshold was 1.0%.

**FIGURE 3:**
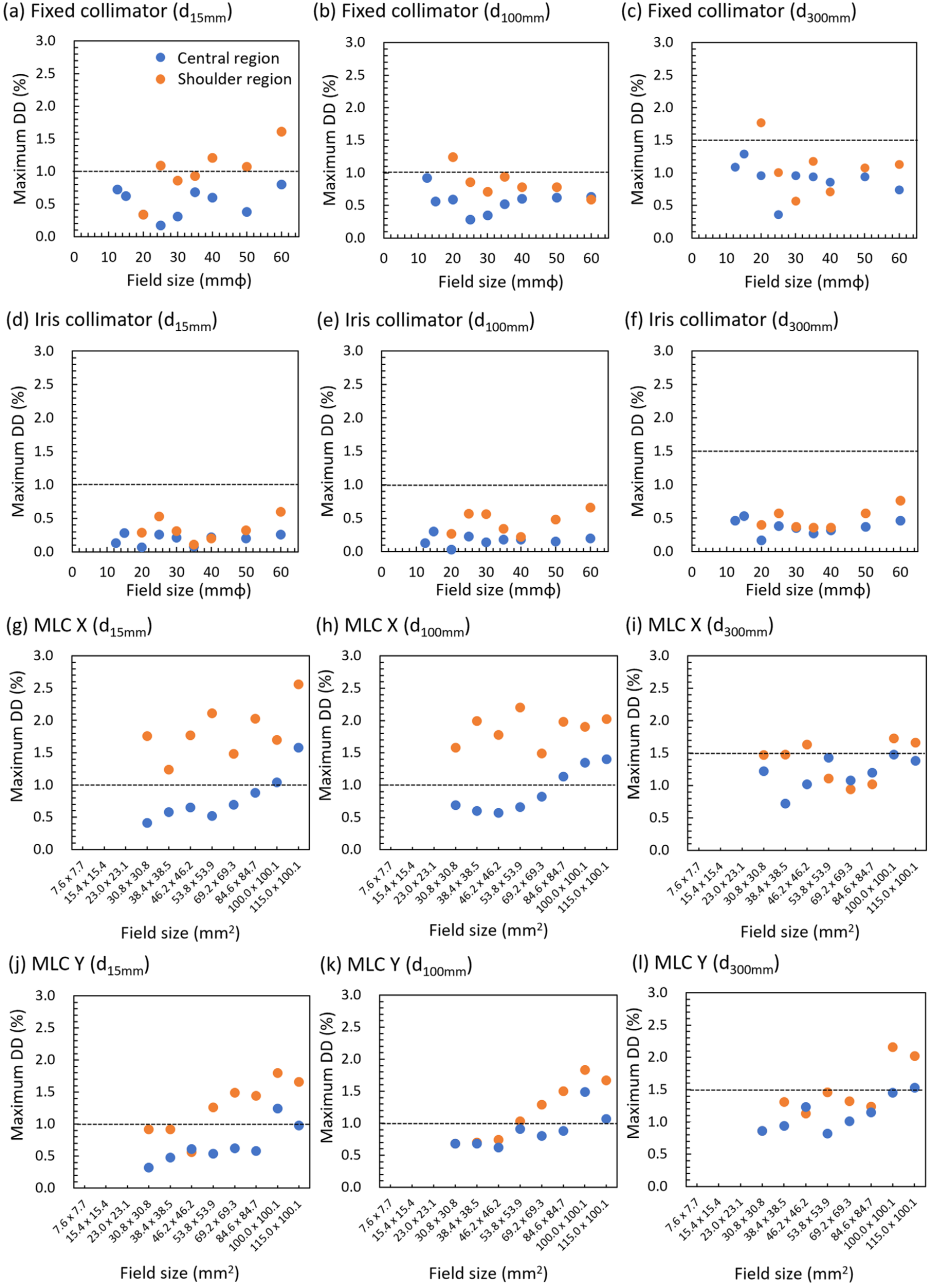
Maximum dose difference (DD) between the vendor supplied beam data (BDM) and measurement beam data in central and shoulder regions for each off‐center ratio (OCR) at depths of 15, 100, and 300 mm (d_15mm_, d_100mm_, and d_300mm_). Dashed lines indicate the thresholds in the central region.

**FIGURE 4:**
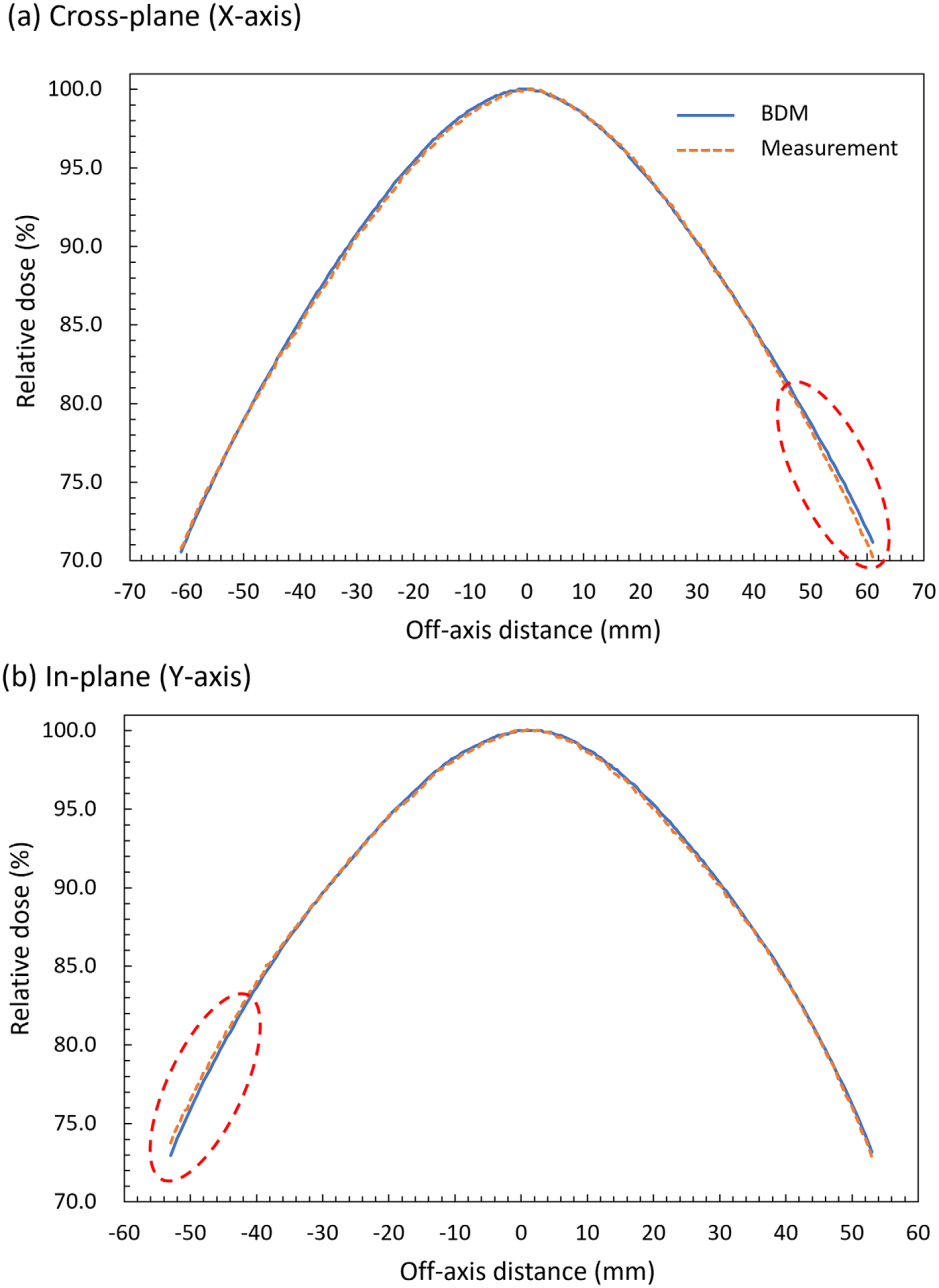
The off‐center ratios (OCRs) of primary beam, excluding secondary collimator, at a depth of 50.0 mm in (a) cross‐plane (X‐axis) and (b) in‐plane (Y‐axis) directions.

**FIGURE 5:**
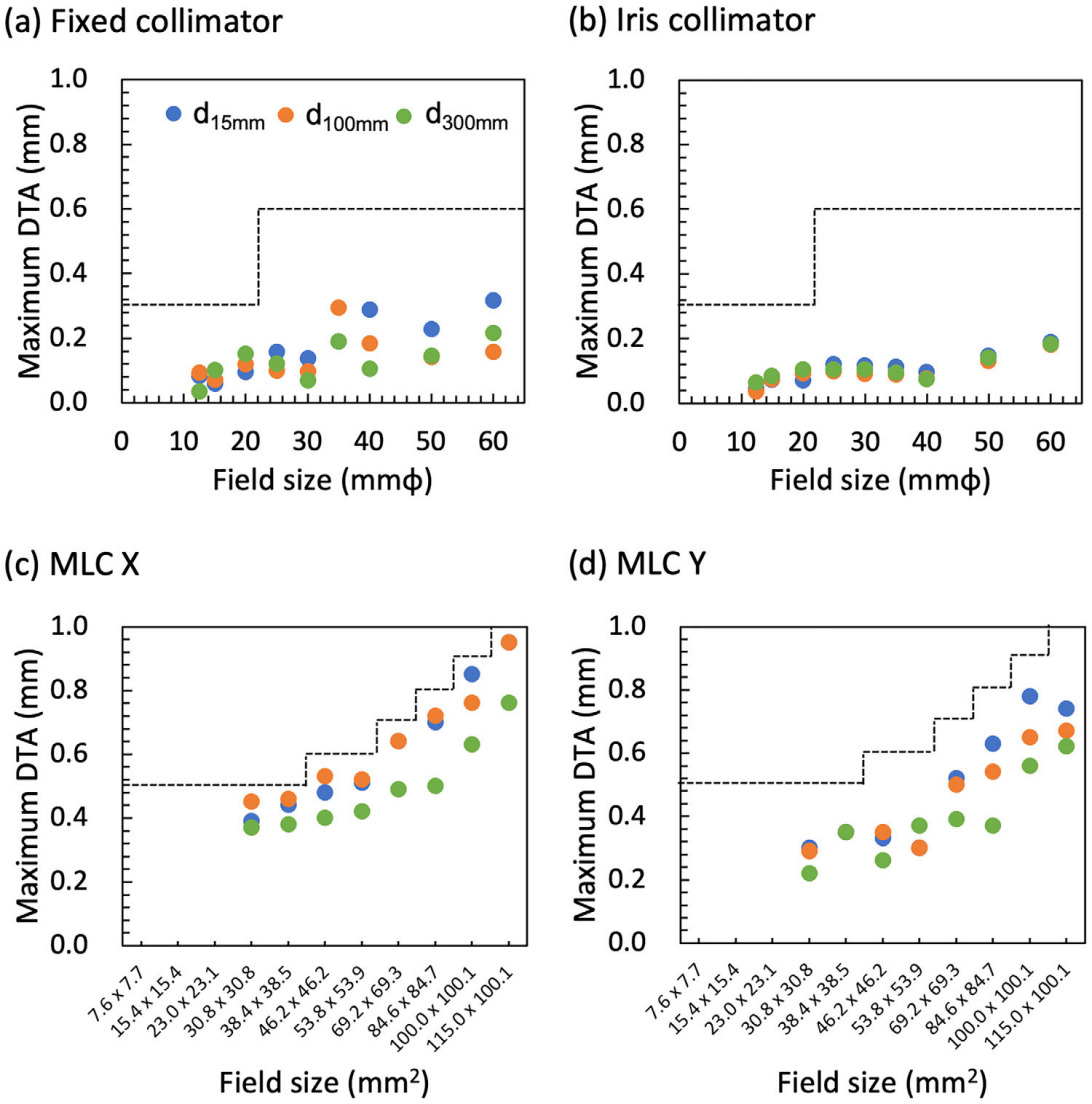
Maximum distance to agreement (DTA) between the vendor supplied beam data (BDM) and measurement beam data in penumbra region for each off‐center ratio (OCR). Dashed lines indicate the thresholds for each field size.

**FIGURE 6:**
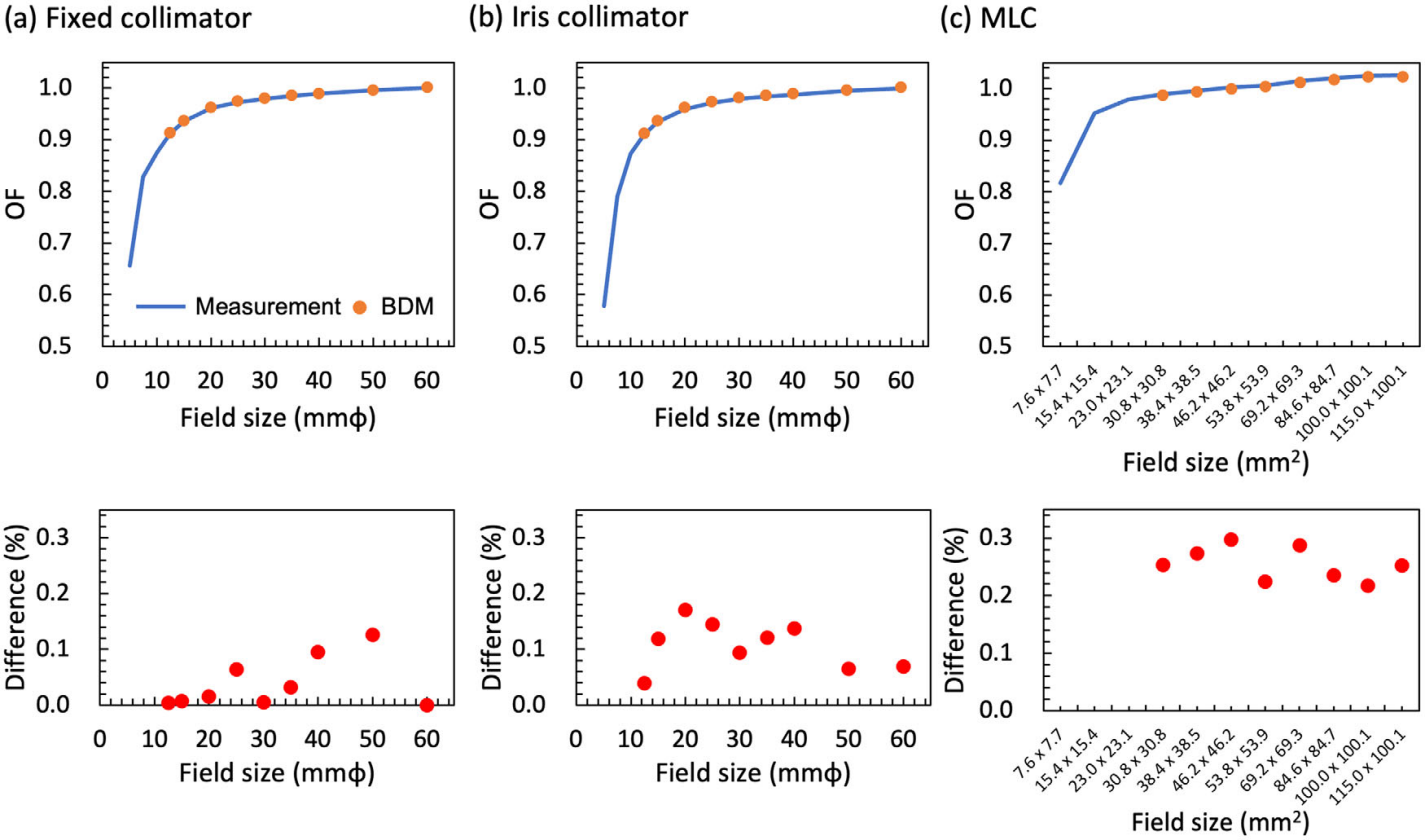
Output factor (OF) and difference between the vendor supplied beam data (BDM) and measurement beam data for each collimator type.

All DDs of the TPRs between the BDM and measurement data were within 1.0% as shown in Figure 2. For the OCRs, the maximum DDs of each collimator and field size are shown in Figure 3. M1 and M2 points for the OCRs of the BDM with fixed collimator, Iris collimator, and MLC are summarized in Tables S2 and S3. All DDs were within 1.0% at d_15mm_ and d_100mm_ and 1.5% at d_300mm_ in the central region, except for the largest three field sizes of the MLC (84.6 × 84.7, 100.0 × 100.1, and 115.0 × 100.1 mm^2^) in Figure 3. The OCRs at a depth of 50.0 mm of primary beam without secondary collimators are also shown in Figure 4. The DDs at off‐axis distances of 50.0 mm and 57.5 mm in the positive direction of the cross‐plane (X‐axis) were 0.4% and 0.7%, respectively, while those at off‐axis distances of 42.3 mm and 50.0 mm in the negative direction of the in‐plane (Y‐axis) were 0.4% and 0.7%, respectively. In Figure 5, the all DTAs were within the thresholds shown in Table 1 for fixed collimator, Iris collimator, and MLC. The OFs are shown in Figure 6 and the differences between the BDM and measurement data were within 0.3%. Then, the h‐BDM was developed according to the above‐mentioned criteria as shown in Table 3.

**TABLE 3:** The hybrid beam data model (h‐BDM) developed by combining of the vendor supplied beam data (BDM) and measurement beam data for use in SRS/SBRT planning.

Fixed / Iris					MLC			
Field size (mmφ)	TPR	OCR	OF		Field size (mm^2^)	TPR	OCR	OF
5.0	Measurement beam data		Measurement beam data		7.6 × 7.7	Measurement beam data		Measurement beam data
7.5					15.4 × 15.4			
10.0					23.0 × 23.1			
12.5	BDM				30.8 × 30.8	BDM		
15.0					38.4 × 38.5			
20.0					46.2 × 46.2			
25.0					53.8 × 53.9			
30.0					69.2 × 69.3			
35.0					84.6 × 84.7			
40.0					100.0 × 100.1	Measurement beam data		
50.0					115.0 × 100.1			
60.0								

### 3.2 Single‐beam validation in homogeneous and heterogeneous phantom geometries

**FIGURE 7:**
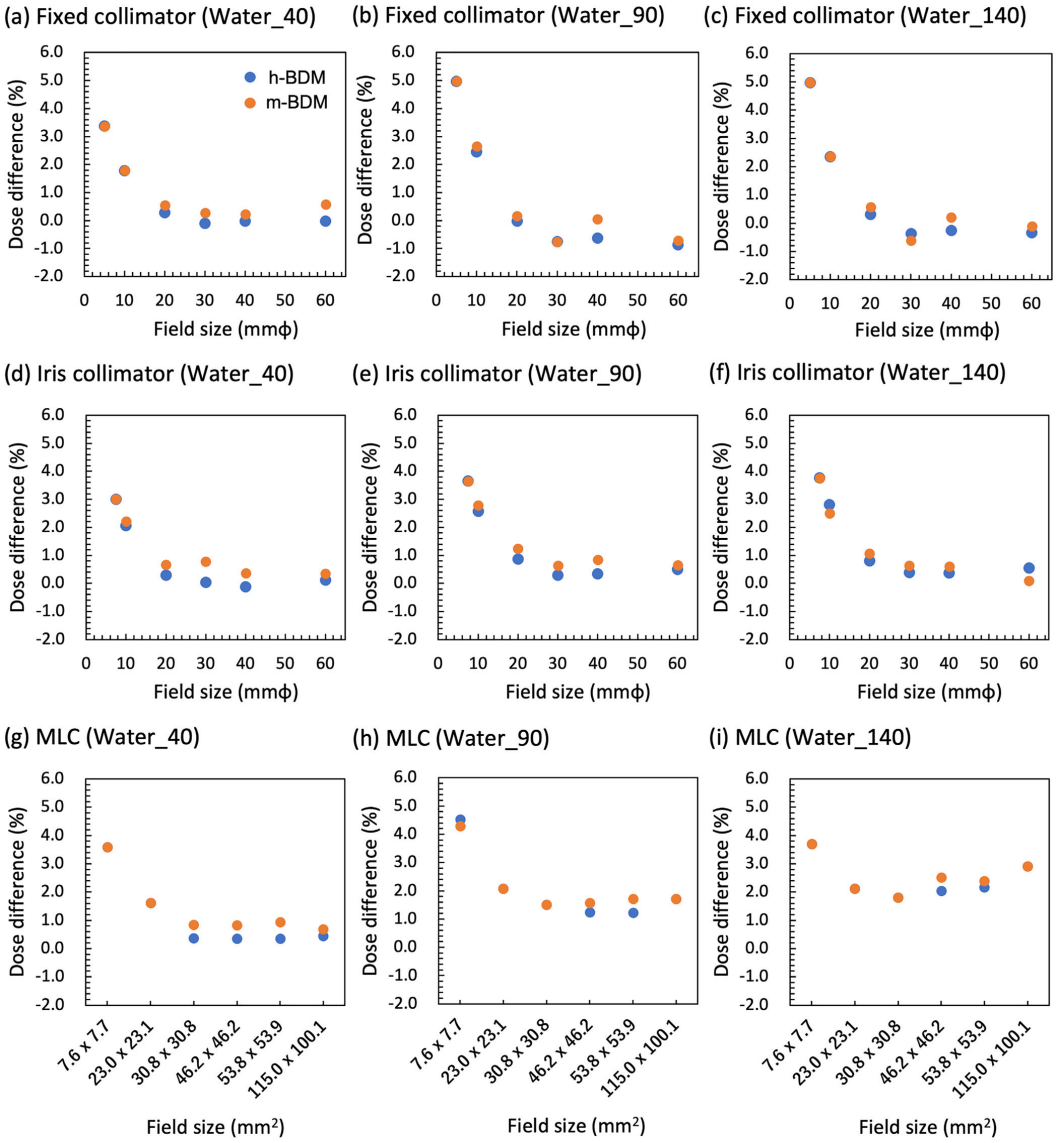
The dose difference (DD) between the measured and calculated doses with the hybrid beam data model (h‐BDM) and the measurement beam data model (m‐BDM) in homogeneous phantom geometry. Positive DD value indicates the calculated dose was higher than the measured dose.

The DDs between the measured and calculated doses with each model using the RT or FSPB algorithm for a single‐beam in homogeneous phantom geometry are shown in Figure 7. For the h‐BDM and m‐BDM, the DDs were within 2.0% for field sizes of > 10.0 mm with each collimator except for Water_140 with the MLC (although the differences were still within 3.0% at this point). The calculated doses with both models for the field sizes of ≤10.0 mm were 3.0–5.0% higher than the measured doses. Across all collimators and field sizes, the calculated DDs between the h‐BDM and the m‐BDM remained within 1.0%.

**FIGURE 8:**
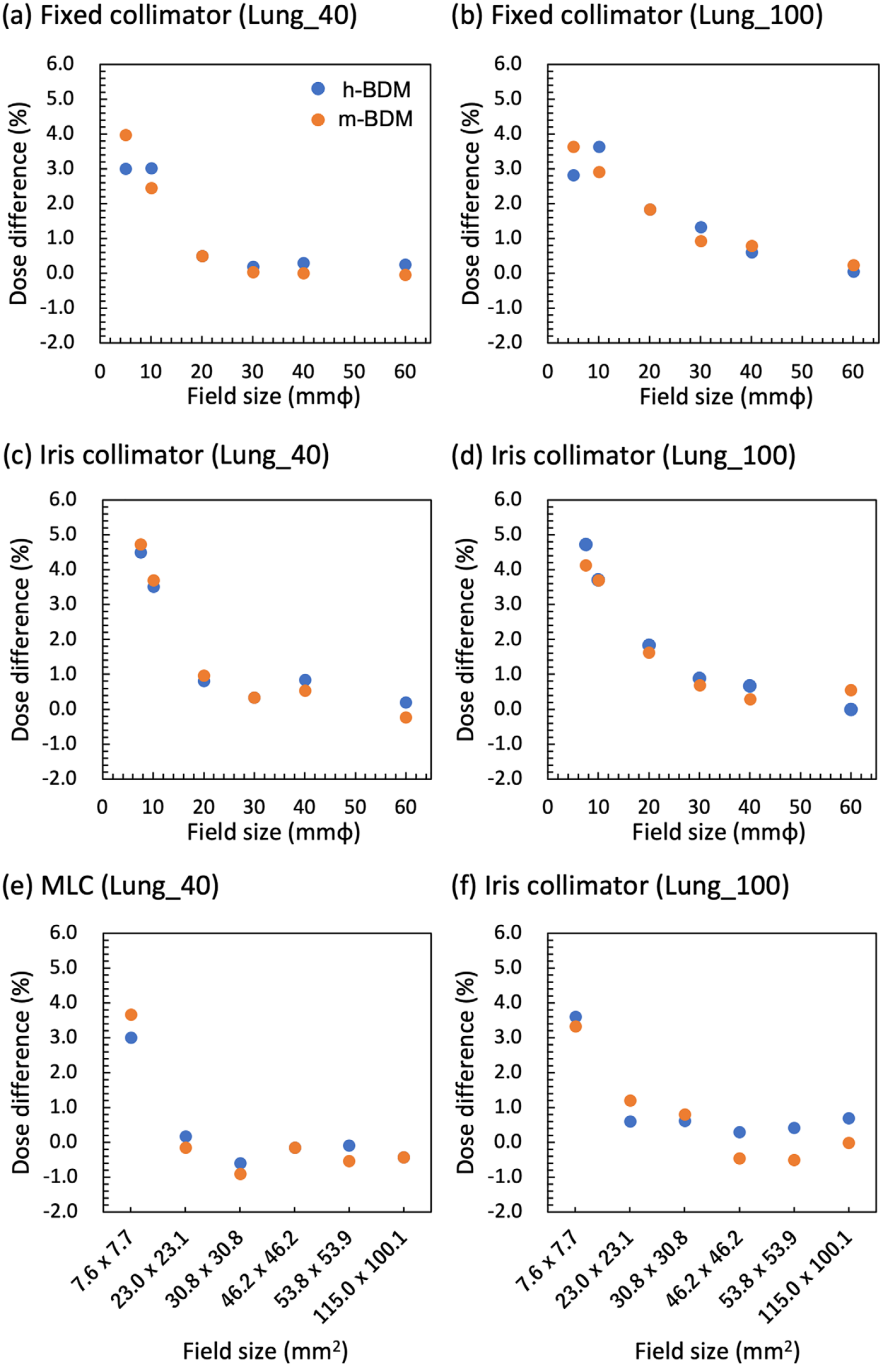
The dose difference (DD) between the measured and calculated doses with the hybrid beam data model (h‐BDM) and the measurement beam data model (m‐BDM) in heterogeneous phantom geometry. Positive DD value indicates the calculated dose was higher than the measured dose.

In heterogeneous phantom geometry, the DDs between the measured and calculated doses with each model using the MC algorithm for a single‐beam are shown in Figure 8. The DDs at the points of Lung_40 and Lung_100 for all models were within 2.0% for field sizes > 10.0 mm with each collimator, except for the 20.0 mmφ beam with the Iris collimator in the h‐BDM at Lung_100, for which it was 2.2%. Similar to the homogeneous geometry results, the calculated doses with both models for field sizes of ≤10.0 mm were 3.0–5.0% higher than the measured doses. All differences between the calculated doses between the h‐BDM and the m‐BDM were within 1.0%.

### 3.3 PSQA of SRS/SBRT plans in phantom and clinical cases

Example dose distributions calculated with the h‐BDM for the SRS/SBRT plans of simulated brain, lung, spine, and prostate in phantom case are shown in Figure S1 and the PSQA results for these cases are summarized in Table 4. All point dose differences between the measured and calculated doses with both models were within 3.0% and all γ pass rates for 2%/2 mm and 3%/1 mm were > 95.0%. The point dose difference between the h‐BDM and m‐BDM was within 1.0% and the γ pass rate differences were within 1.0% (2%/2 mm and 3%/1 mm) and 2.2% (2%/1 mm).

**TABLE 4:** The patient‐specific quality assurance (PSQA) results of the point dose difference and the γ pass rate for the hybrid beam data model (h‐BDM) and the measurement beam data model (m‐BDM) in phantom case. Positive point dose difference value indicates the calculated dose with each model was higher than the measured dose.

			γ pass rate (%)		
Case	Model	Point dose difference (%)	2% / 2 mm	3% / 1 mm	2% / 1 mm
Brain 10.0 mmφ (Fixed Collimator)	h‐BDM	1.8	99.0	99.8	98.1
	m‐BDM	1.7	99.4	99.6	98.4
Brain 20.0 mmφ (Fixed Collimator)	h‐BDM	0.8	99.7	99.8	98.1
	m‐BDM	1.8	99.8	99.6	98.4
Brain 30.0 mmφ (Fixed Collimator)	h‐BDM	0.9	100.0	100.0	100.0
	m‐BDM	1.8	100.0	100.0	100.0
Brain 10.0 mmφ (Iris Collimator)	h‐BDM	1.8	99.5	100.0	99.0
	m‐BDM	2.0	99.5	100.0	98.2
Brain 20.0 mmφ (Iris Collimator)	h‐BDM	−0.3	99.9	100.0	99.7
	m‐BDM	−0.9	100.0	100.0	99.9
Brain 30.0 mmφ (Iris Collimator)	h‐BDM	−1.0	100.0	100.0	100.0
	m‐BDM	−1.5	100.0	100.0	100.0
Lung 10.0 mmφ	h‐BDM	1.9	99.0	100.0	98.5
	m‐BDM	1.5	100.0	100.0	100.0
Lung 20.0 mmφ	h‐BDM	0.6	100.0	100.0	99.9
	m‐BDM	0.5	100.0	100.0	100.0
Lung 30.0 mmφ	h‐BDM	−0.2	99.5	99.9	98.9
	m‐BDM	−0.4	99.2	99.7	98.1
C‐Shape (AAPM TG119)	h‐BDM	1.0	99.4	99.5	94.6
	m‐BDM	1.6	99.2	98.5	92.7
Test Prostate (AAPM TG119)	h‐BDM	2.1	98.8	98.6	92.8
	m‐BDM	2.6	99.4	99.5	95.0

In the clinical case, examples of the dose distributions calculated with the h‐BDM in the SRS/SBRT plans for the brain, lung, spine, and prostate are shown in Figure S2 and the corresponding PSQA results are summarized in Table 5. All point dose differences between the measured and calculated doses with both models were within 3.0%, and all of the γ pass rates for 2%/2 mm and 3%/1 mm were > 95.0%. The point dose difference between the h‐BDM and m‐BDM was within 0.4% and the γ pass rate differences were within 1.2% (2%/2 mm and 3%/1 mm) and 1.9% (2%/1 mm). As shown in Table 3, the BDM for the three largest MLC field sizes were additionally replaced with measured beam data; therefore, spine and prostate SBRT plans using the MLC were created with the pure h‐BDM, and PSQA was performed. For the spine SBRT plan, the point dose difference was 0.5%, and the γ pass rates were 97.9%, 97.7%, and 89.1% for the 2%/2 mm, 3%/1 mm, and 2%/1 mm criteria, respectively. For the prostate SBRT plan, the point dose difference was 2.4%, and the γ pass rates were 97.5%, 98.7%, and 90.7%, respectively. The point dose differences were within 3.0%, and the γ pass rates for the 2%/2 mm and 3%/1 mm criteria were > 95.0% for both plans. Compared with the m‐BDM, the point dose differences were within 0.5% for both plans. All differences in γ pass rates were within 1.0% for the prostate SBRT plan, whereas the γ pass rates with the pure h‐BDM were lower than those with the m‐BDM by 1.7% (2%/2 mm and 3%/1 mm) and by 7.5% (2%/1 mm) in the spine SBRT plan.

**TABLE 5:** The patient‐specific quality assurance (PSQA) results of the point dose difference and the γ pass rate for the hybrid beam data model (h‐BDM) and the measurement beam data model (m‐BDM) in clinical case. Positive point dose difference value indicates the calculated dose with each model was higher than the measured dose.

			γ pass rate (%)		
Case	Model	Point dose difference (%)	2%/2 mm	3%/1 mm	2%/1 mm
Brain (PitNET)	h‐BDM	2.5	99.8	98.9	97.8
	m‐BDM	2.5	99.5	97.7	96.3
Brain (Metastasis)	h‐BDM	−0.8	99.6	99.9	99.2
	m‐BDM	−1.2	99.6	99.9	99.2
Lung (Size of 19.0 mm)	h‐BDM	−1.4	99.5	99.5	96.4
	m‐BDM	−1.3	99.3	99.3	96.2
Lung (Size of 38.0 mm)	h‐BDM	−2.0	96.1	97.4	90.3
	m‐BDM	−2.2	95.5	97.9	89.3
Spine	h‐BDM	0.9	98.4	98.6	94.7
	m‐BDM	0.9	99.6	99.4	96.6
Prostate	h‐BDM	2.6	97.9	98.6	90.9
	m‐BDM	2.9	97.5	99.1	91.5

## 4 DISCUSSION

This study is the first clinical validation of the CyberComm commissioning process for the CyberKnife system. We demonstrated that a clinically acceptable SRS/SBRT commissioning process can be achieved using the h‐BDM, which was developed by selective incorporation of measurement beam data into the novel BDM. To develop the h‐BDM, the basic BDM validation was firstly implemented, although the all TPRs and OCRs satisfied the acceptance criteria for the vendor's beam twinning test with the γ pass rates.^10^, ^11^ Because the γ pass rate lacks sensitivity to the magnitude of discrepancies between the BDM and measurement, the thresholds for each DD and DTA were proposed in this study, as shown in Table 1. The thresholds of DD were set to 1.0% for TPR and OCRs at d_15mm_ and d_100mm_ which is typically considered a clinically acceptable level, while a threshold of 1.5% was applied to OCRs at d_300mm_ to account for measurement uncertainty. The DTA criterion for the OCRs followed the vendor's recommendation used in the gamma index analysis.^10^, ^11^ The differences between BDM and measurement exceeded the proposed thresholds for the three largest field sizes with MLC for OCRs as shown in Figure 3 g–l. This might be affected by the differences between the BDM and measurement at the OCR edges of the primary beam, as shown in Figure 4, and the positional uncertainty of the MLC. Thus, the h‐BDM was developed by replacement of the BDM with the measurement beam data as shown in Table 3.

For the modeling validation, dosimetric accuracy of the calculated doses with the h‐BDM were evaluated with single‐beam in homogeneous and heterogeneous geometries. The AAPM TG 135.B recommends the use of ionization chamber for the PSQA.^15^ The RAZOR ion‐chamber was employed without applying small‐field correction factors to maintain the consistency from the single‐beam validation to the PSQA. The DDs between the measured and calculated doses were within 2.0% for the field sizes of > 10.0 mm and within 5.0% for the field sizes of ≤10.0 mm with each collimator as shown in Figures 7 and 8. Those differences were within the tolerance limits recommended by the AAPM TG 135.B and MPPG 9.b. that the relative OF differences between the measurements and calculations were within 2.0% with field sizes of > 10.0 mm and 3.0% as acceptable or 5.0% as action limits for the field sizes of ≤10.0 mm.^15^, ^28^ The differences for the extremely small field sizes can be attributed to the high uncertainty, the difficulty of the measurements, and the inherent tendency toward underestimation characteristic of the RAZOR ion‐chamber, which is similar to the predecessor CC01 ion‐chamber (IBA Dosimetry, Schwarzenbruck, Germany) as reported in IAEA TRS‐483.^29^ This indicates that the DDs for the field sizes of ≤10.0 mm may be mitigated. Another study has also described the differences between the measured and calculated doses were increased with increasing the depth. The DDs using collapsed cone convolution algorithm, which was comparable calculation accuracy with RT and FSPB algorithms, were within 2.0% to the depth of 90.0 mm for field size of 38.0 × 60.0 mm^2^ with the MLC, while up to 3.2% at depth of 195.0 mm.^30^ Furthermore, compared to the calculated dose with the conventional m‐BDM, the differences with the h‐BDM were within 1.0%. Thus, the beam modeling with the h‐BDM in this study was equivalent to the previously reports and considered as clinically acceptable.

Finally, the SRS/SBRT plan deliverability with the h‐BDM was validated by the PSQA in phantom and clinical cases. The point dose differences between the measurements and calculations were within 3.0%, and the γ pass rates were > 95.0% for 2%/2 mm and 3%/1 mm criteria for all PSQA of the SRS/SBRT plans, as shown in Tables 4 and 5. Those results were considered as clinical acceptable based on the tolerance limits set by the AAPM TG 135.B and MPPG 9.b of the point dose differences of 5.0% and γ pass rates of 90.0% for 2%/2 mm and 3%/1 mm.^15^, ^28^ The differences between the BDM and measurement beam data for the largest MLC field sizes exceeded the thresholds given in Table 1 as a result of the factor illustrated in Figure 4. To evaluate the effect of replacing these beam data with the user‐measured beam data, spine and prostate SBRT plans using the MLC were created with the pure h‐BDM, and the PSQA results of these plans were compared with those obtained using the conventional m‐BDM. The relatively large difference observed with the strict criterion in the spine SBRT plan may be attributed to the difference in beam data exceeding the thresholds; however, the pure h‐BDM may have potential for clinical use according to the AAPM TG 135.B and MPPG 9.b.^15^, ^28^ The thresholds proposed in this study should be considered preliminary. Multicenter studies are needed to establish the optimal thresholds through post hoc analyses.

Beam data in this study were measured during a new installation of the CyberKnife system. The CyberComm approach, using the vendor‐supplied BDM, may reduce the burden on users and provide them with greater reassurance. The measurement of extremely small field beams is highly difficult, and there is considerable inter‐unit variation; however, through the optimal use of the BDM in the preconfigured CyberKnife system, the potential setup and measurement errors and the commissioning times and workload could be dramatically reduced. A spot check for the intermediate field sizes, the combination of the BDM and measurement data for the OFs, and the standardization of institutional beam data are also expected. Further multicenter validation studies are warranted to demonstrate the usefulness and generalizability of the vendor‐supplied BDM.

## 5 CONCLUSIONS

The practical method of the h‐BDM development is the supplementation of the BDM with measurement beam data for all OFs and TPRs and OCRs in the three smallest field sizes and in the cases where the differences between the BDM and measurement exceed the following thresholds: DD of 1.0% for TPR, DD of 1.0% at d_15mm_ and d_100mm_ and of 1.5% at d_300mm_ in the OCR central region, and DTA of 0.3‐1.0 mm in the OCR penumbra region. The SRS/SBRT commissioning process with this proposed h‐BDM provides a new clinically acceptable framework.

## AUTHOR CONTRIBUTIONS

Conceptualization and Methodology: Mikoto Tamura, Yasumasa Nishimura, Seiki Nishino, Hirokazu Mizuno, Iori Sumida, and John M. Noll. Measurements: Mikoto Tamura, Yuta Inosaka, Yoshiyuki Sadahira, Ayano Akamine, and Kyomi Inoue. Treatment planning: Mikoto Tamura, Yasumasa Nishimura, and Tomohiro Matsuura. Data analysis: Mikoto Tamura and Yoshiyuki Sadahira. Resources: Hirokazu Mizuno and Iori Sumida. Manuscript preparation: Mikoto Tamura, Yasumasa Nishimura, Tomohiro Matsuura, Seiki Nishino, Hirokazu Mizuno, Iori Sumida, and John M. Noll. All authors read and approved the final manuscript.

## CONFLICT OF INTEREST STATEMENT

The authors declare that they received financial support from Accuray Inc. for covering the manuscript proofreading and publication fees. No other conflicts of interest are reported.

## ETHICS APPROVAL STATEMENT

The Institutional Ethics Committee approved this study (institutional review board number: 2024018).

## Supporting information




**Table S1**. Prescribed dose and dose constraints of organs at risk (OARs) in SRS/SBRT plans in clinical case.

**Table S2**. M1 and M2 values for the off‐center ratios (OCRs) of the vendor supplied beam data (BDM) with fixed and Iris collimators.

**Table S3**. M1 and M2 values for the off‐center ratios (OCRs) of the vendor supplied beam data (BDM) with MLC.

**Supporting Information**: acm270822‐sup‐0004‐Figure S1.tif

**Supporting Information**: acm270822‐sup‐0005‐Figure S2.tif

## Data Availability

The data supporting the findings of this study are available from the corresponding author upon reasonable request.
