# Supplementary material for: SRS/SBRT commissioning process with a hybrid beam data model in new preconfigured CyberKnife system
Source: J Appl Clin Med Phys. 2026 Sep 25;27(10):e70822. doi: 10.1002/acm2.70822 (PMC13615346; doi:10.1002/acm2.70822)
Supplement: Supplementary file 1 [file ACM2-27-e70822-s001.docx]

Table S1. Prescribed dose and dose constraints of organs at risk (OARs) in SRS/SBRT plans in clinical case.

| Brain | | |  | Lung | |
| --- | --- | --- | --- | --- | --- |
|  | PitNET | Metastasis |  |  |  |
| PTV | D_99.5_ = 25 Gy/5 fr | D_99.5_ = 24 Gy/3 fr |  | PTV | D_95_ = 48 Gy/4 fr |
| Normal brain  (Brain – GTV) | V_25Gy_ < 16.0 cc | V_18Gy_ < 26.0 cc |  | Normal lung  (Lung – GTV) | D_mean_ < 18.0 Gy |
|  | V_28.8Gy_ < 7.0 cc | V_21Gy_ < 21.0 cc |  |  | V_15Gy_ < 25.0% |
|  |  | V_24Gy_ < 16.8 cc |  |  | V_20Gy_ < 20.0% |
| Brain stem | D_max_ < 31.0 Gy | D_max_ < 23.1 Gy |  |  | V_40Gy_ < 100.0 cc |
|  | V_23Gy_ < 0.5 cc | V_15.9Gy_ < 0.5 cc |  | Spinal cord | D_max_ < 23.0 Gy |
| Optic pathway | D_max_ < 25.0 Gy | D_max_ < 17.4 Gy |  | Trachea | D_max_ < 34.8 Gy |
|  | V_22Gy_ < 0.35 cc | V_15.3Gy_ < 0.2 cc |  | Esophagus | D_max_ < 30.0 Gy |
| Cochlea | D_max_ < 22.0 Gy | D_max_ < 14.4 Gy |  | Brachial plexus | D_max_ < 26.0 Gy |
| Spine | | |  | Prostate | |
| PTV | D_95_ = 35 Gy/5 fr | |  | PTV | D_95_ = 36.25 Gy/5 fr |
| Spinal cord | D_max_ < 25.3 Gy | |  | Rectum | V_18.1Gy_ < 50.0% |
|  |  |  |  |  | V_29Gy_ < 20.0% |
| Esophagus | D_max_ < 35.0 Gy | |  |  | V_36Gy_ < 1.0 cc |
|  |  |  |  | Bladder | V_18.1Gy_ < 40.0% |
| Trachea | D_max_ < 35.0 Gy | |  |  | V_37Gy_ < 5.0 cc |
|  |  |  |  | Urethra | V_42Gy_ < 50.0% |
