# Supplementary material for: SRS/SBRT commissioning process with a hybrid beam data model in new preconfigured CyberKnife system
Source: J Appl Clin Med Phys. 2026 Sep 25;27(10):e70822. doi: 10.1002/acm2.70822 (PMC13615346; doi:10.1002/acm2.70822)
Supplement: Supplementary file 2 [file ACM2-27-e70822-s003.docx]

Table S2. M1 and M2 values for the off-center ratios (OCRs) of the vendor supplied beam data (BDM) with fixed and Iris collimators.

| Depth (mm) | Field size (mmφ) | Fixed | | Iris | |
| --- | --- | --- | --- | --- | --- |
|  |  | M1 (mm) | M2 (mm) | M1 (mm) | M2 (mm) |
| 15 | 12.5 | 3.88 | 8.53 | 3.73 | 8.68 |
| 100 |  | 3.73 | 8.68 | 3.58 | 8.88 |
| 300 |  | 3.48 | 8.93 | 3.33 | 9.13 |
| 15 | 15.0 | 5.13 | 9.88 | 4.98 | 9.93 |
| 100 |  | 4.98 | 10.03 | 4.83 | 10.13 |
| 300 |  | 4.78 | 10.28 | 4.63 | 10.38 |
| 15 | 20.0 | 7.78 | 12.43 | 7.48 | 12.43 |
| 100 |  | 7.68 | 12.58 | 7.33 | 12.58 |
| 300 |  | 7.48 | 12.78 | 7.18 | 12.78 |
| 15 | 25.0 | 10.23 | 15.08 | 9.83 | 14.98 |
| 100 |  | 10.18 | 15.18 | 9.73 | 15.08 |
| 300 |  | 10.03 | 15.38 | 9.58 | 15.33 |
| 15 | 30.0 | 12.83 | 17.68 | 12.33 | 17.48 |
| 100 |  | 12.83 | 17.78 | 12.23 | 17.58 |
| 300 |  | 12.68 | 17.93 | 12.08 | 17.83 |
| 15 | 35.0 | 15.43 | 20.28 | 14.73 | 19.93 |
| 100 |  | 15.33 | 20.38 | 14.58 | 20.08 |
| 300 |  | 15.18 | 20.53 | 14.48 | 20.28 |
| 15 | 40.0 | 17.98 | 22.83 | 17.13 | 22.38 |
| 100 |  | 17.88 | 22.93 | 17.03 | 22.58 |
| 300 |  | 17.73 | 23.08 | 16.88 | 22.78 |
| 15 | 50.0 | 23.18 | 27.93 | 22.08 | 27.43 |
| 100 |  | 22.98 | 28.08 | 21.93 | 27.68 |
| 300 |  | 22.83 | 28.28 | 21.78 | 27.93 |
| 15 | 60.0 | 28.43 | 33.08 | 27.03 | 32.53 |
| 100 |  | 28.18 | 33.28 | 26.88 | 32.78 |
| 300 |  | 28.03 | 33.43 | 26.73 | 33.03 |
