# Supplementary material for: SRS/SBRT commissioning process with a hybrid beam data model in new preconfigured CyberKnife system
Source: J Appl Clin Med Phys. 2026 Sep 25;27(10):e70822. doi: 10.1002/acm2.70822 (PMC13615346; doi:10.1002/acm2.70822)
Supplement: Supplementary file 3 [file ACM2-27-e70822-s005.docx]

Table S3. M1 and M2 values for the off-center ratios (OCRs) of the vendor supplied beam data (BDM) with MLC.

| Depth (mm) | Field size (mm^2^) | MLC X | | MLC Y | |
| --- | --- | --- | --- | --- | --- |
|  |  | M1 (mm) | M2 (mm) | M1 (mm) | M2 (mm) |
| 15 | 30.8 × 30.8 | 12.93 | 17.73 | 12.08 | 17.58 |
| 100 |  | 12.83 | 17.88 | 11.93 | 17.68 |
| 300 |  | 12.73 | 18.13 | 11.73 | 17.88 |
| 15 | 38.4 × 38.5 | 16.73 | 21.53 | 15.93 | 21.53 |
| 100 |  | 16.63 | 21.68 | 15.78 | 21.48 |
| 300 |  | 16.48 | 21.93 | 15.58 | 21.68 |
| 15 | 46.2 × 46.2 | 20.63 | 25.43 | 19.78 | 25.13 |
| 100 |  | 20.48 | 25.63 | 19.63 | 25.33 |
| 300 |  | 20.38 | 25.83 | 19.38 | 25.58 |
| 15 | 53.8 × 53.9 | 24.43 | 29.23 | 23.68 | 29.03 |
| 100 |  | 24.28 | 29.43 | 23.53 | 29.18 |
| 300 |  | 24.08 | 29.68 | 23.33 | 29.43 |
| 15 | 69.2 × 69.3 | 32.08 | 36.93 | 31.38 | 36.73 |
| 100 |  | 31.93 | 37.08 | 31.18 | 36.88 |
| 300 |  | 31.83 | 37.33 | 31.03 | 37.08 |
| 15 | 84.6 × 84.7 | 39.83 | 44.58 | 39.18 | 44.43 |
| 100 |  | 39.73 | 44.73 | 39.03 | 44.58 |
| 300 |  | 39.63 | 44.93 | 38.83 | 44.78 |
| 15 | 100.0 × 100.1 | 47.63 | 52.18 | 46.93 | 52.13 |
| 100 |  | 47.48 | 52.43 | 46.73 | 52.33 |
| 300 |  | 47.38 | 52.58 | 46.63 | 52.43 |
| 15 | 115.0 × 100.1 | 55.13 | 59.73 | 46.93 | 52.13 |
| 100 |  | 54.98 | 59.93 | 46.73 | 52.13 |
| 300 |  | 54.83 | 60.08 | 46.58 | 52.43 |
